# Early Age Carbonation Heat and Products of Tricalcium Silicate Paste Subject to Carbon Dioxide Curing

**DOI:** 10.3390/ma11050730

**Published:** 2018-05-04

**Authors:** Zhen Li, Zhen He, Yixin Shao

**Affiliations:** 1 State Key Laboratory of Water Resources and Hydropower Engineering Science, Wuhan University, Wuhan 430072, China; lizhen2012@whu.edu.cn; 2 Department of Civil Engineering and Applied Mechanics, McGill University, 817 Sherbrooke Street West, Montreal, QC H3A 2K6, Canada; yixin.shao@mcgill.ca

**Keywords:** carbonation heat, carbonation curing, CO_2_ uptake, TEM-EDS, ^29^Si MAS NMR

## Abstract

This paper presents a study on the carbonation reaction heat and products of tricalcium silicate (C_3_S) paste exposed to carbon dioxide (CO_2_) for rapid curing. Reaction heat was measured using a retrofitted micro-calorimeter. The highest heat flow of a C_3_S paste subject to carbonation curing was 200 times higher than that by hydration, and the cumulative heat released by carbonation was three times higher. The compressive strength of a C_3_S paste carbonated for 2 h and 24 h was 27.5 MPa and 62.9 MPa, respectively. The 24-h carbonation strength had exceeded the hydration strength at 28 days. The CO_2_ uptake of a C_3_S paste carbonated for 2 h and 24 h was 17% and 26%, respectively. The X-ray diffraction (XRD), transmission electron microscope coupled with energy dispersive spectrometer (TEM-EDS), and ^29^Si magic angle spinning–nuclear magnetic resonance (^29^Si MAS-NMR) results showed that the products of a carbonated C_3_S paste were amorphous silica (SiO_2_) and calcite crystal. There was no trace of calcium silicate hydrate (C–S–H) or other polymorphs of calcium carbonate (CaCO_3_) detected.

## 1. Introduction

There is an increasing interest in the early age carbonation curing of Portland cement in recent years for durability improvement [1,2] and carbon dioxide (CO_2_) utilization [3,4,5,6,7]. Since tricalcium silicate (C_3_S) is the major phase in Portland cement, accounting for 50% by weight, understanding the reaction mechanism of C_3_S exposed to CO_2_ activation will be of great importance to promote commercial applications of the technology.

Carbonation reaction is a strong exothermic reaction. The calculated reaction heat for C_3_S carbonation was about 347.4 kJ/mol [8]. It is believed that the rapid strength gain due to carbonation reaction and the formation of carbonation products are attributed to the carbonation reaction heat. However, there is no report on carbonation heat from the carbonation reaction of C_3_S, which is due possibly to a lack of proper testing methods.

In the 1970s, Berger et al. [9,10,11] had studied the effect of the water to binder ratio, CO_2_ pressure, and carbonation time on the strength development of C_3_S mortar. It was found that low-lime calcium silicate hydrate (C–S–H) and calcite were the main reaction products. Goto et al. [12] used thermogravimetric analysis (TG), gas phase mass spectroscopy, and X-ray diffraction (XRD) to examine the carbonation products of C_3_S. The results showed that an amorphous calcium silicate hydrocarbonate was generated along with the calcium carbonate (CaCO_3_). Shtepenko et al. [13] used ^29^Si magic angle spinning–nuclear magnetic resonance (^29^Si MAS-NMR), XRD and a scanning electron microscope coupled with an energy dispersive spectrometer (SEM-EDS) to analyze the carbonation of β-dicalcium silicate (β-C_2_S), and concluded that polymerized silica, calcite, and aragonite were the main products. Ashraf and Olek [14] believed that Ca-modified silica gel and CaCO_3_ crystal were the main products of C_3_S carbonation. From the above studies, it was indicative that the carbonation products of C_3_S could be classified into two phases: silica phase and calcium carbonate phase. The forms of silica phase found by different studies could be low-lime C–S–H, calcium silicate hydrocarbonate, or polymerized silica (SiO_2_) gel. For the CaCO_3_ phase, all of the polymorph forms of calcite, aragonite, and vaterite were observed.

There are discrepancies in reports on carbonation products. This can be attributed to a number of reasons. The first one can be the carbonation conditions. For example, the CO_2_ used in the study of Berger et al. was pure, while in the study of Goto et al., it was 5% purity. Meanwhile, the gas pressures also play a role. Most of the results were obtained from the carbonation of powder samples. The second reason is the limit of test methods. The XRD method commonly used in the above research is suited for the crystal phase, but is not effective for the non-crystal phase. The ^29^Si MAS NMR method used in the study of Shtepenko et al. can better explore the structure of silicon phase in amorphous products. The TG method can accurately reveal the detail of carbonation products, especially for the quantity analysis of CaCO_3_. In the study of Shtepenko et al., the SEM-EDS technique was used to test the Ca/Si ratio in the micro-area; however, the space resolution of SEM-EDS is limited. So, the statistic results based on SEM-EDS may have some deviation.

The objective of this paper is to measure the early age carbonation heat of C_3_S paste and determine the carbonation products of C_3_S paste by different test methods. A retrofitted micro-calorimeter was used to obtain the data of heat release by injecting CO_2_ gas into the reaction cell. The transmission electron microscope coupled with energy dispersive spectrometer (TEM-EDS) method, which had a higher spatial resolution [15], was employed for microstructure analysis. Thermogravimetric analysis–derivative thermogravimetric analysis (TG-DTG), XRD, and ^29^Si magic angle spinning–nuclear magnetic resonance (^29^Si MAS-NMR) were also applied to investigate the carbonation products of C_3_S paste.

## 2. Materials and Methods

**Figure 1:**
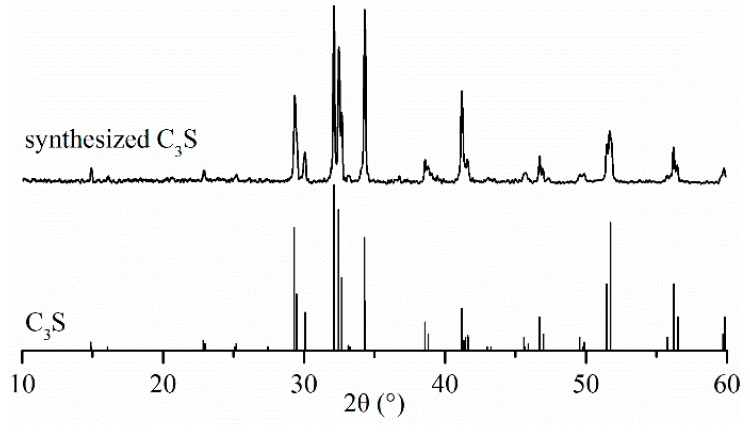
X-ray diffraction (XRD) pattern of synthesized C_3_S.

C_3_S was synthesized by method of solid phase sintering [16]. It was prepared using analytical reagent calcium hydroxide (Ca(OH)_2_) and SiO_2_ with molar ratios of 3:1. The mixtures of Ca(OH)_2_ and SiO_2_ were mixed with a ball mill for 8 h to ensure they were evenly mixed. After that, a small amount of anhydrous ethanol was added to the mixture and compressed into cakes. The sintering processes were implemented as following: the compressed cakes were gradually heated from room temperature to 1000 °C over a period of 100 min; the temperature was kept at 1000 °C for 60 min; then, the temperature increased from 1000 °C to 1550 °C; and was then kept at 1550 °C for 4 h. The sintering process was repeated as described above, until the content of free-CaO was negligible. The synthesized C_3_S was ground to powder with a Blaine specific surface area of 380 m^2^∙kg^−1^ in a planetary mill (Pulverisette 7 Premium Line, FFRITSCH, Idar-Oberstein, Rhineland-Palatinate, Germany). The polymorph of synthesized C_3_S is M_3_, which was determined by XRD [17,18], and its pattern is shown in Figure 1. Its chemical compositions, as determined by X-ray fluorescence (XRF) analysis, are presented in Table 1.

**Figure 2:**
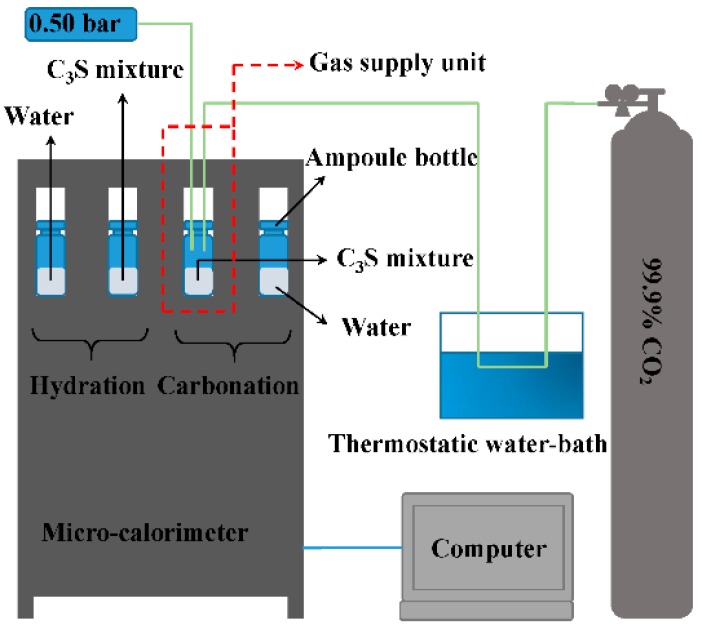
Schematic diagram of carbonation and hydration reaction heat experimental setup.

**Figure 3:**
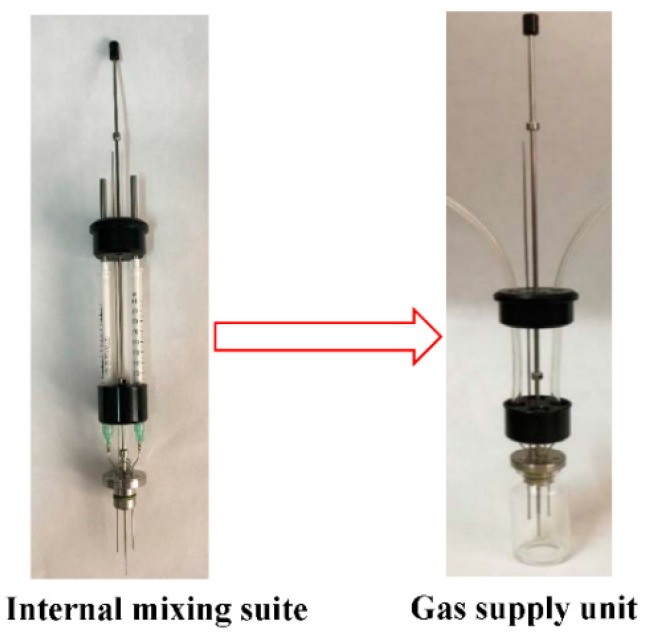
Photo of gas supply unit retrofitted by internal mixing suite.

Heat flow and the amount of released heat during carbonation and hydration were measured using a retrofitted micro-calorimeter (TAM AIR, TA, New Castle, DE, USA). The testing temperature was set at 20 °C. A schematic diagram of the carbonation and hydration reaction heat experimental setup is shown in Figure 2. The C_3_S paste was prepared by mixing C_3_S powder and water with a water-to-binder ratio (w/b) of 0.15. This ratio has been proved to be optimum by other studies [7,10,19]. A C_3_S mixture of 5 g was loaded into an ampoule bottle. For hydration, the sample was sealed immediately and loaded into the micro-calorimeter. A certain amount of water with the same heat capacity was used as the reference sample. For carbonation, an internal mixing suite (an accessory of the micro-calorimeter) was retrofitted to a gas supply unit; CO_2_ gas could enter into the ampoule through the pipe. A photo of the gas supply unit is shown in Figure 3. Constant temperature was achieved using a thermostatic water bath. Through earlier trials, it was observed that water at 68 °C could keep the incoming gas at a steady temperature of 20 °C. This ensured that the incoming gas would not affect the micro-calorimetric measurement. The internal pressure of the ampoule bottle was maintained at 0.5 bar to avoid very high pressure, which could lead to the explosion of the ampoule bottle. The data of heat flow and heat were recorded using a calorimeter continuously for 60 h.

**Figure 4:**
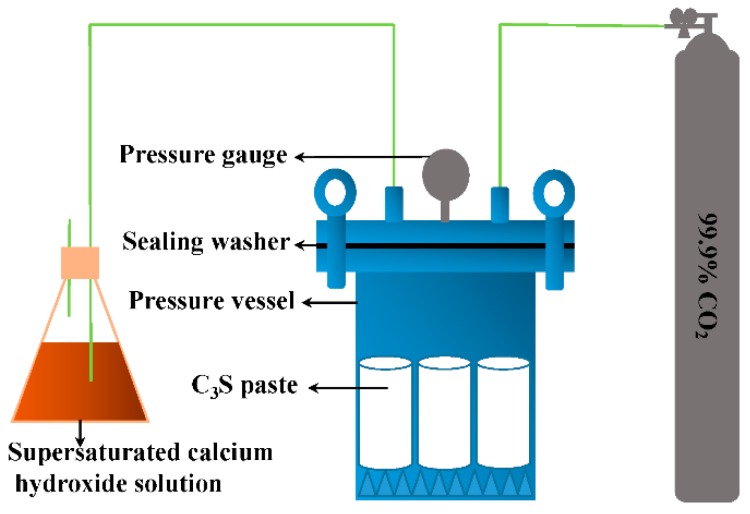
Schematic diagram of the carbonation curing setup.

For the compressive strength test and other micro-analysis tests: 25 g of C_3_S mixture with 0.15 w/b was loaded into a stainless steel mold, and pressed with 8 MPa pressure to make it into a cylindrical sample with the size of ϕ20 × 40 mm. The molding pressure of 8 MPa proved to be the most favorable for carbonation reaction [19,20]. The demolded samples were cured following the curing details shown in Table 2. The hydration samples of H2h, H24h, and H28d were loaded into a curing chamber immediately. The hydration curing conditions were relative humidity (RH) ≥ 95% and 20 °C. Samples of H2h, H24h, and H28d were cured for 2 h, 24 h, and 28 days, respectively. Figure 4 shows the schematic diagram of a carbonation curing setup. The carbonation curing conditions were as follows: the purity of CO_2_ was 99.9%; the curing pressure was 4 bar; the test ambient temperature was 20 ± 3 °C; and RH ≥ 75%. The carbonation samples were named C2h and C24h, according to the different curing time. In order to determine the maximum possible CO_2_ uptake of C_3_S paste under the experimental conditions, a batch of RC (repeated carbonation) was investigated. The samples of RC were directly carbonated for four times without compaction for 24 h. Between different carbonation processes, the sample was ground, dried, and then mixed with water at 0.15 w/b again. A compressive strength test was then carried out on the samples that were cured to the specified age. The crushed samples were collected and put into anhydrous alcohol to stop the further reaction. Then, the collected crushed samples were further milled, dried at 60 °C, and sealed for TG-DTG, XRD, TEM-EDS, and ^29^Si MAS NMR tests.

The theoretical CO_2_ uptake is calculated by Steinour’s equation, as shown in Equation (1). Since C_3_S was a pure phase without influence by SO_3_, Na_2_O, and K_2_O, the CO_2_ uptake capacity was calculated by CaO content, which was determined by XRF. The experimental CO_2_ uptake is calculated by TG data, the calculation equation is shown in Equation (2). In Equation (2), the sample weight is the total weight of the tested samples. The total weight loss is the loss of samples from room temperature to 1000 °C. The weight loss of decarbonation is the mass loss between 500 and 1000 °C. The carbonation degree of the sample is calculated using Equation (3):

Theoretical CO_2_ uptake (%) = 0.785 (CaO − 0.7 SO_3_) + 1.09 Na_2_O + 0.93 K_2_O, (1)

Experimental CO_2_ uptake (%) = (weight loss of decarbonation)/(sample weight − total weight loss) × 100%, (2)

Carbonation degree (%) = (experimental CO_2_ uptake)/(theoretical CO_2_ uptake) × 100%. (3)

Q600 (TA, New Castle, DE, USA) was used to obtain the TG-DTG results; the range of the machine was 200 mg. The weight of the tested sample was about 30 mg. The heating rate was 10 °C/min from room temperature to the final 1000 °C. N_2_ was adopted as the protecting gas with a flow rate of 100 mL/min. A D8 ADVANCE (Bruker, Karlsruhe, Baden-Wurttemberg, Germany) with a Cu Kα target, a scanning range of 15–55°, and a scanning speed of 2°/min was utilized for the XRD test. The TEM-EDS result was tested by Tecnai G2 F30 transmission electron microscope (FEI, Hillsborough, OR, USA) and its attached energy dispersive spectrometer. ^29^Si MAS NMR experiments were performed using a AVANCE III 600 spectrometer (Bruker, Karlsruhe, Baden-Wurttemberg, Germany) at a resonance frequency of 119.2 MHz. The spectra was recorded on a 4-mm probe with a spinning rate of 10 kHz, a π/4 pulse length of 2.6 μs, and a recycle delay of 80 s. The chemical shifts of ^29^Si was referenced to tetramethylsilane.

## 3. Results

### 3.1. Carbonation Reaction Heat

**Figure 5:**
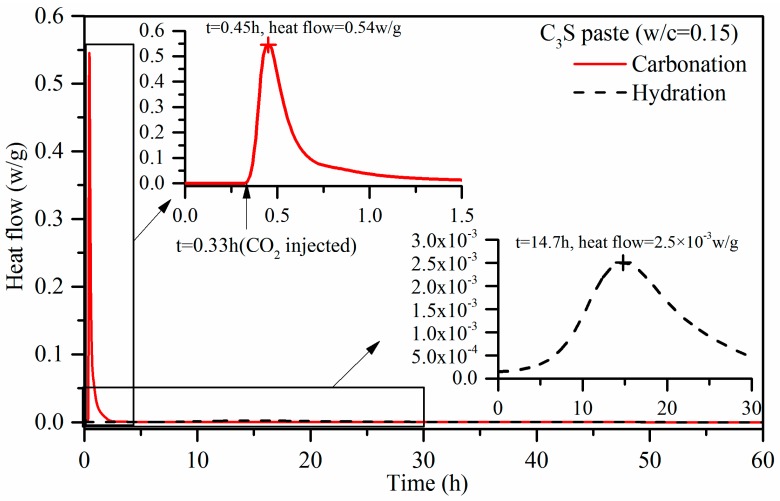
Heat flow of carbonation and hydration versus time.

Figure 5 shows the heat flow of carbonation and hydration versus time. From the partially enlarged detail, it can be seen that the heat flow increased significantly at the moment of the injection of CO_2_, and reached the peak in a few minutes. This suggested that the carbonation reaction of C_3_S paste occurs immediately upon the exposure to the carbon dioxide gas under the conditions of 0.15 w/b, 0.5 bar pressure, and 99.9% purity of CO_2_. It was observed that the peak value of carbonation heat flow was up to 0.54 W/g, after which it fell rapidly in approximately 10 minutes, and continued to fall gradually to a lower value. The reason for the decrease in heat flow could be the lack of water. This was evident by a large amount of condensed water on the wall of the ampule bottle after the test. For the partially enlarged detail of the hydration curve, it was seen that the heat flow increased very slowly. It took approximately 15 h to reach the peak. The maximum heat flow of hydration was less than 1% in comparison to that generated by carbonation.

**Figure 6:**
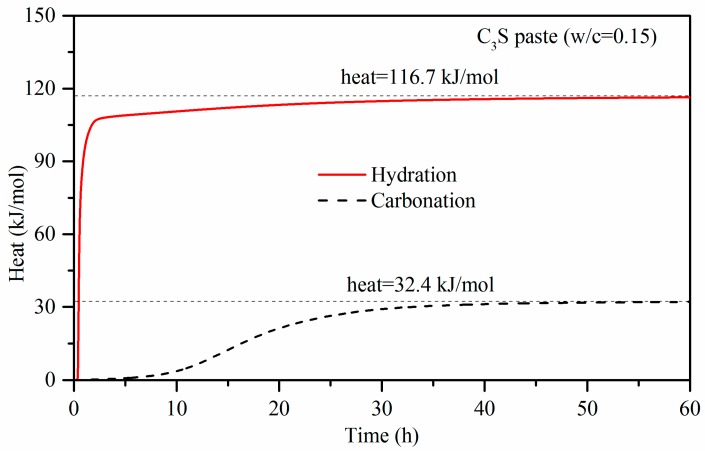
Heat of carbonation and hydration versus time.

Figure 6 shows the heat of carbonation and hydration versus time. Due to the higher heat flow, the heat rapidly reached a high value in the carbonation sample. In 60 h, the carbonation heat of C_3_S was up to 116.7 kJ/mol. Although the heat release for hydration took longer, the hydration heat of hydration was only 32.4 kJ/mol, which is about one third that of carbonation.

### 3.2. Compressive Strength

**Figure 7:**
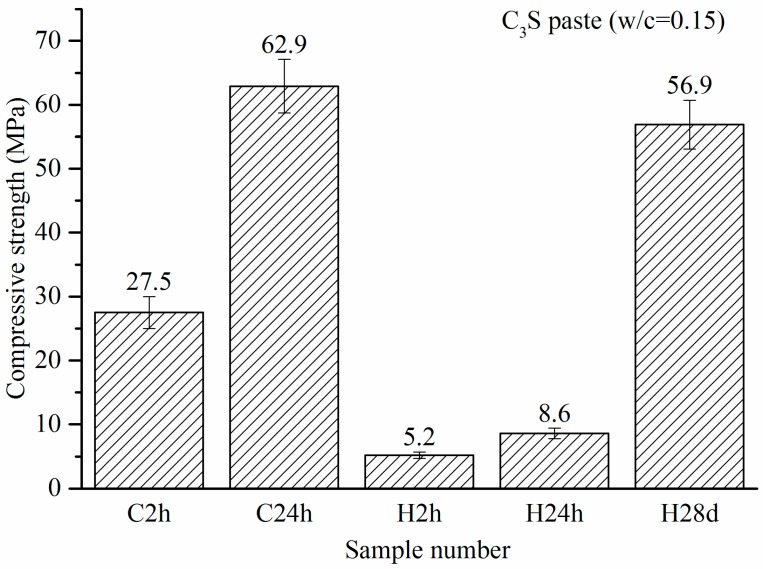
Compressive strengths of carbonation and hydration C_3_S pastes.

The compressive strengths of the carbonation and hydration C_3_S pastes are shown in Figure 7. It was seen that the compressive strength of the C_3_S paste reached 27.5 MPa after 2 h of carbonation. In comparison, the hydration reference sample had shown a strength of 5.2 MPa, which was attributed to the compression molding instead of the hydration reaction. The compressive strength of the C_3_S paste carbonated for 24 h (C24h) reached 62.9 MPa. Meanwhile, the compressive strength of H24h, which hydrated for 24 h, was only 8.6 MPa, which was much lower than that of the carbonated sample. The strength of C24h was even higher than that of the samples hydrated for 28 days (H28d), which was 56.9 MPa. The comparison of C2h and C24h showed that the strength gain was proportional to the carbonation time. The early carbonation of C_3_S is an accelerated reaction process due to the rapid gain in strength. Therefore, the carbonation curing can be used to shorten the production cycle and gain economic benefits.

### 3.3. Carbonation Depth

**Figure 8:**
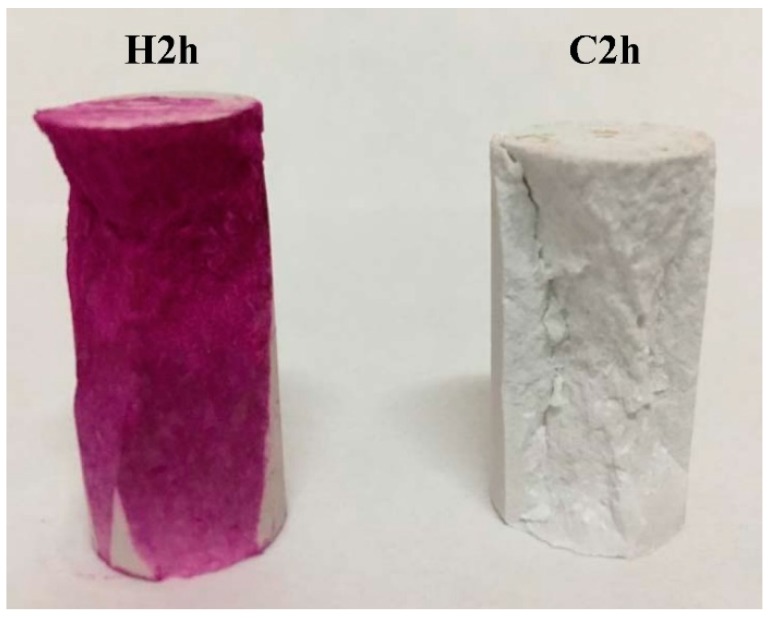
Samples sprayed by an alcohol phenolphthalein solution.

Pictures of cracked samples sprayed with alcohol phenolphthalein solution are presented in Figure 8. The hydration sample turned red because of the alkaline Ca(OH)_2_ that was generated by the hydration reaction. The carbonation sample was colorless throughout entire cross-section, suggesting that carbonation had penetrated to the entire specimen in 2 h.

### 3.4. TG-DTG

**Figure 9:**
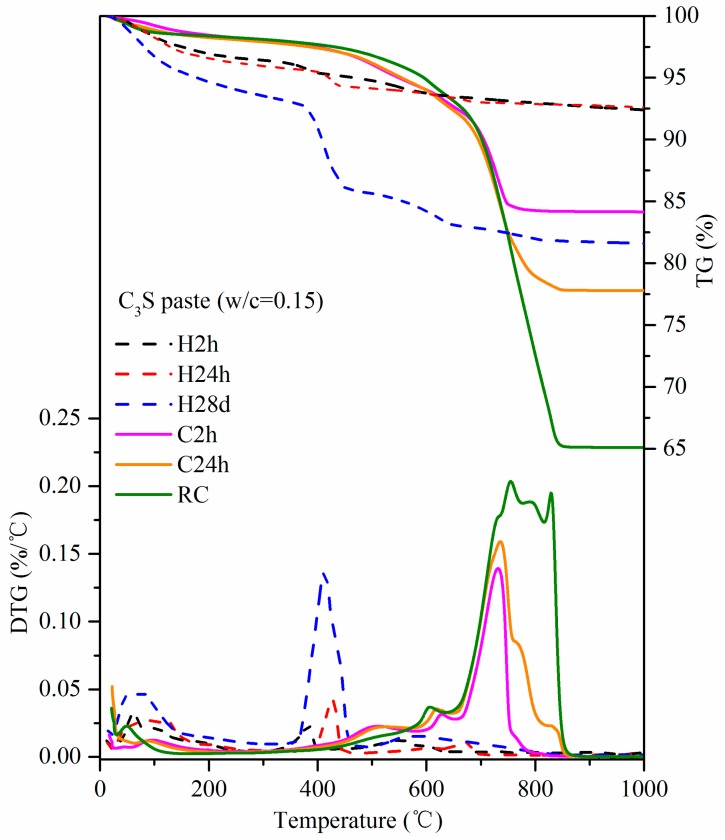
Thermogravimetric analysis–derivative thermogravimetric analysis (TG-DTG) curves of carbonation and hydration samples.

The TG-DTG curves of carbonation and hydration samples are shown in Figure 9. The weight loss before 105 °C was due to the evaporation of free water. The weight loss between 105 °C and 300 °C was due to the dehydration of C–S–H. The weight loss between 300–500 °C was caused by dehydroxylation. In this study, the main reason was the dehydroxylation of Ca(OH)_2_. The weight loss above 500 °C was due to the decomposition of CaCO_3_ [21,22,23]. In the dehydration temperature range of C–S–H (105–300 °C), the weight loss of the three hydration batches was obvious. The weight loss increased with the hydration time, suggesting that the longer the hydration time, the greater amount of C–S–H. However, for carbonation batches in the same temperature range (105–300 °C), there was almost no C–S–H due to the low weight loss shown in Figure 9. The peak of weight loss for Ca(OH)_2_ dehydroxylation was obvious at about 400 °C in the hydration sample. However, the peak did not exist in the carbonation sample in the same range. The peak of weight loss due to CaCO_3_ decomposition was observed obviously in carbonation samples. The TG curves showed that the weight loss increased with the increase of the carbonation time in the carbonation samples. Since the batch of RC had been added with water and repeatedly carbonized, its degree of reaction was highest. Hence, the weight loss of RC was greatest. This indicated that in addition to the short carbonation time, the lack of water was the other factor limiting further carbonation. From the DTG curve, it was seen that the peak temperature of the decomposition of CaCO_3_ increased with the increase of carbonation; most of the CaCO_3_ decomposed after 750 °C, especially in the RC sample. This was related to the amount of CaCO_3_ and the degree of crystallization. The greater the amount of CaCO_3_ and the higher degree of crystallization, the higher the decomposition temperature.

The results regarding weight loss, CO_2_ uptake, and carbonation degree are shown in Table 3. The CO_2_ uptake of C2h and C24h was 17.17% and 26.32%, respectively. The observation of CO_2_ uptake increased with the increase of carbonation time, which was consistent with the conclusions of other studies [5,6,7]. The CO_2_ uptake of RC had reached 51.11%. Although it was of little significance for practical use, the results revealed the maximum possible CO_2_ uptake capacity of the C_3_S paste exposed to CO_2_.

### 3.5. XRD

**Figure 10:**
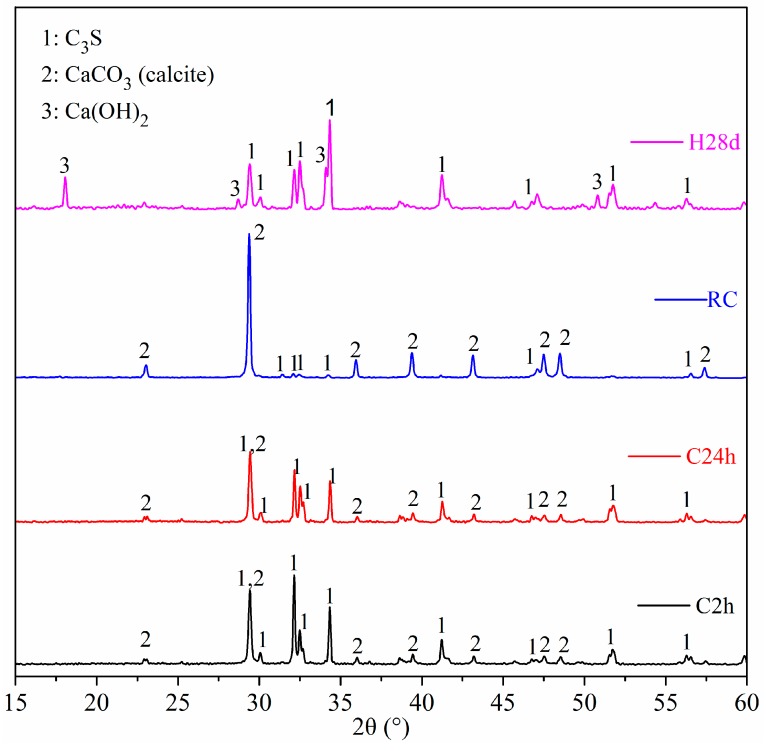
XRD pattern of carbonation and hydration samples.

XRD test was carried out to identify the crystalline phase in the product. The XRD patterns are shown in Figure 10. The result showed that the unreacted C_3_S and Ca(OH)_2_ were the main crystalline phases in the hydration sample. While in the carbonation samples, CaCO_3_ and unreacted C_3_S were the main crystalline phases. No other polymorphs appeared, except for calcite in the carbonation sample. The intensity of the calcite diffraction peak increased, while that of the C_3_S decreased with the prolonged carbonation time. This suggested that the carbonation process consumed C_3_S and simultaneously generated calcite. The diffraction peak of C_3_S in RC was very weak, but the peak of calcite was very high. This showed that the carbonation degree of RC was high. The degree of carbonation reaction reflected by the XRD results was consistent with TG-DTG.

### 3.6. TEM-EDS

**Figure 11:**
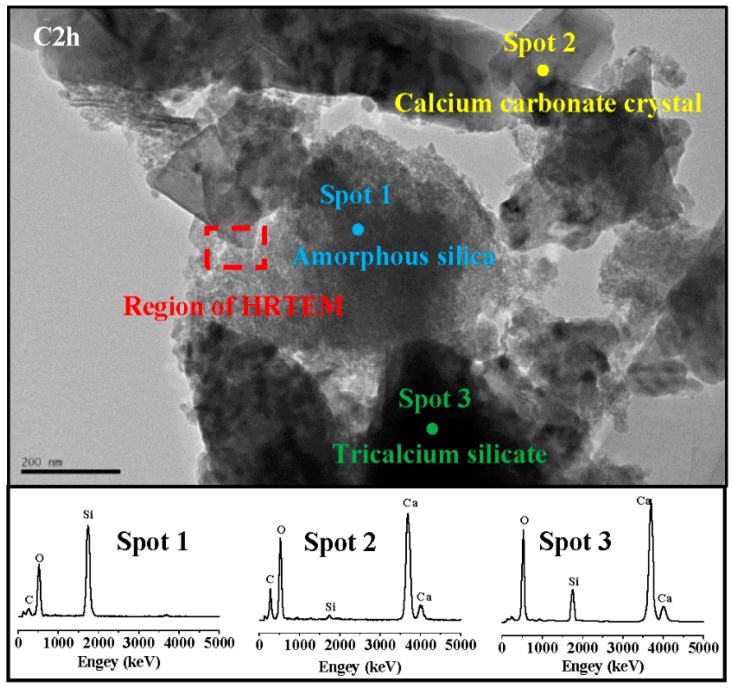
Transmission electron microscope (TEM) image and EDS mappings of C2h.

**Figure 12:**
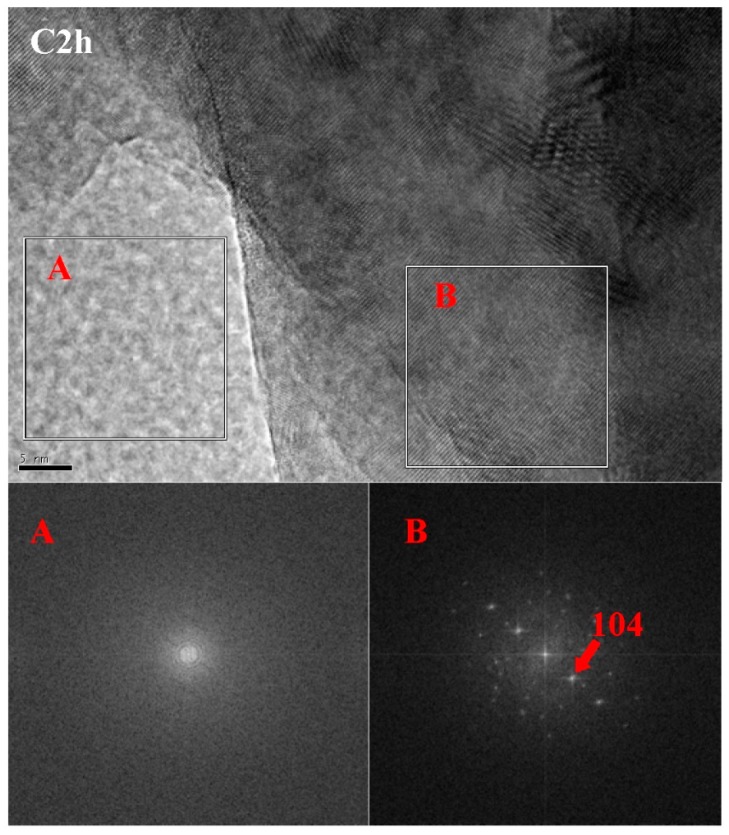
High-resolution TEM (HRTEM) image and selected area Fourier transform images of C2h.

Figure 11 is a TEM image and energy dispersive spectrum (EDS) mappings of C2h. According to the morphology of phase, there were mainly three phases: a cloud phase (Spot 1), a crystal phase (Spot 2) and a darker lump phase (Spot 3). The atomic Si:O in the cloud phase was close to 1:2. The diffraction peaks of Si were not found in the former XRD result, suggesting that the cloud phase was amorphous silica [24,25]. The EDS results showed that there was no Si in the crystal phase, confirming that the crystal phase was CaCO_3_. The dark lump was unreacted C_3_S, which was confirmed by the EDS result of Ca:Si, which had an atomic ratio close to 3:1. Figure 12 shows a high-resolution TEM (HRTEM) image and a selected area Fourier transform image of C2h. The diffraction rings in A and the diffraction lattice in B were respectively correspondent with the amorphous SiO_2_ and crystal CaCO_3_. It was also observed that the amorphous SiO_2_ was close to the crystal CaCO_3_, and had a clear interface.

**Figure 13:**
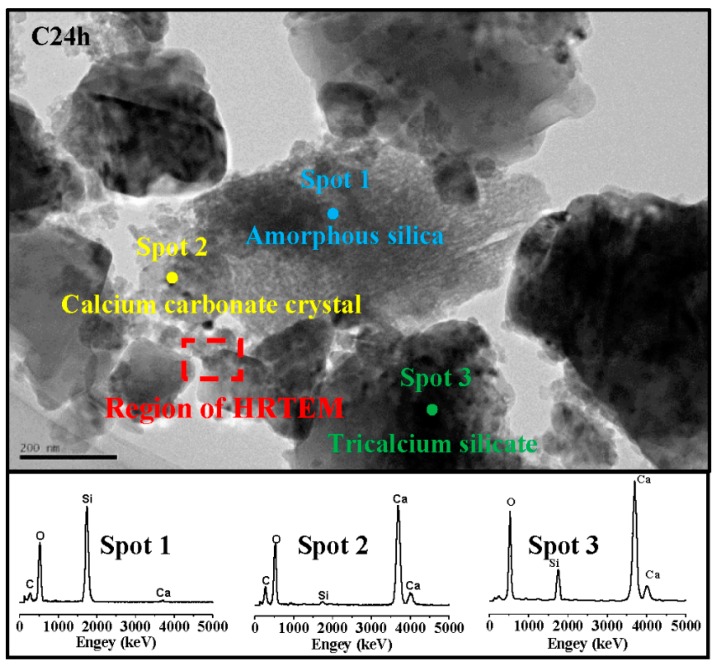
TEM image and EDS mappings of C24h.

**Figure 14:**
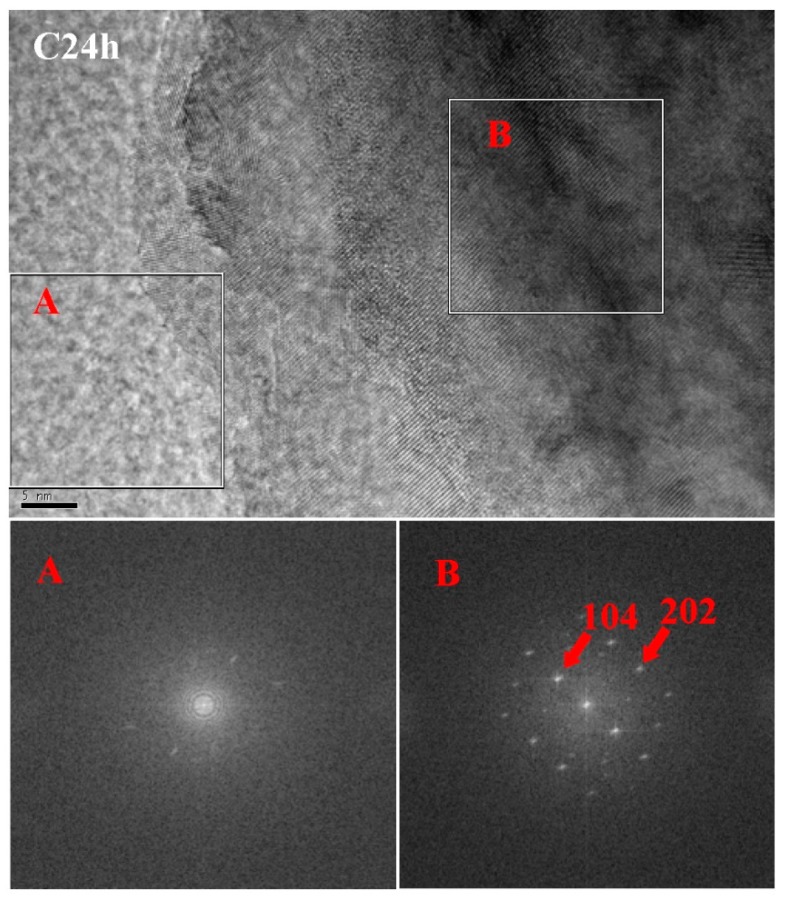
HRTEM image and selected area Fourier transform images of C24h.

A TEM image and EDS mappings of C24h are also present in Figure 13. The HRTEM image and selected area Fourier transform images of C24h are presented in Figure 14. The C24h sample had no new phase compared with C2h, according to the phase morphology in Figure 11. It was difficult to find the relative content difference of the product phases due to the small visual field. The figures also show that the microstructures in nanoscale were similar at 2 h and 24 h of carbonation, although the latter produced higher strength and higher CO_2_ uptake, which were also evident by the TG-DTG and XRD results.

### 3.7. ^29^Si MAS NMR

**Figure 15:**
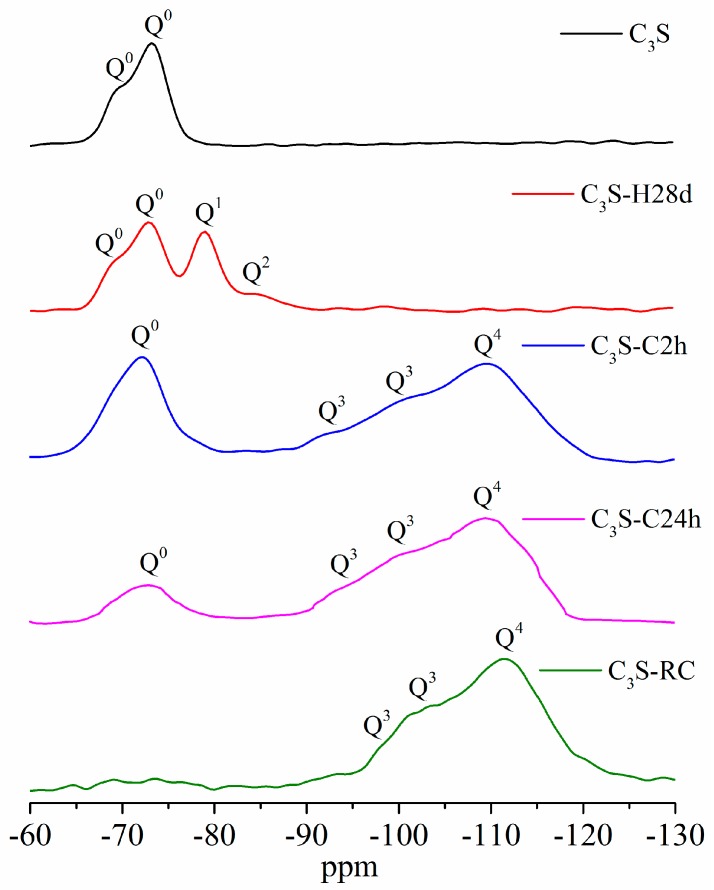
^29^Si magic angle spinning–nuclear magnetic resonance (MAS-NMR) pattern of different samples.

The ^29^Si MAS NMR spectra of different samples are shown in Figure 15. The resonance in this figure was divided into five ranges: −65 to −75 ppm belonged to Q^0^; near −80 ppm belonged to Q^1^; near −85 ppm belonged to Q^2^; near −100 ppm belonged to Q^3^; and near −110 ppm belonged to Q^4^ [26,27,28]. The resonance peak of Q^0^ originated from silicate monomer, and in this study, it came from unreacted C_3_S. The resonance peaks of Q^1^ and Q^2^ originated from the end and middle of the C–S–H chain structure. The resonance peaks of Q^3^ and Q^4^ originated from the chain-branching sites and three-dimensional cross-linked framework [29], respectively. These peaks came from amorphous SiO_2_ in this study.

There were only Q^0^ resonance peaks in the sample of synthesized C_3_S. After 28 days of hydration, the intensity of Q^0^ decreased, while the intensity of the Q^1^ and Q^2^ that originated from C–S–H increased simultaneously. The carbonation samples mainly had the resonance peaks of Q^0^ of unreacted C_3_S, and Q^3^ and Q^4^ of amorphous SiO_2_. No resonance peaks of Q^1^ and Q^2^ from C–S–H appeared in carbonation samples. It showed that there was few or no C–S–H in carbonation samples. The intensity of Q^0^ decreased with the progress of carbonation. It showed that unreacted C_3_S reduced as carbonation time increased. This result was consistent with XRD. The intensity of Q^3^ and Q^4^ increased with the increase of carbonization time. At the same time, Q^3^ had the tendency to transform into Q^4^.

## 4. Discussion

The experimental carbonation heat of C_3_S was 116.7 kJ/mol, which was measured by micro- calorimeter, and is lower than the calculation value 347.4 kJ/mol in the research of Goodbrake et al. [8]. As mentioned in the former text, the lack of water inhibited the carbonation reaction. The main reason for the experimental carbonation heat being lower than the calculation value is the incomplete carbonation reaction.

The results of reaction heat and TG-DTG show that the rate and degree of the carbonation reaction were higher than those of the hydration reaction. The high heat flow increased the reaction temperature in a short time, and the hydrolysis of C_3_S and the speed of the diffusion of calcium ions were both accelerated by the high reaction temperature. Compared with the hydration of C_3_S paste, the formation of Ca(OH)_2_ was substituted by CaCO_3_ in the carbonation reaction. Due to the low solubility of CaCO_3_, more calcium ions were dissolved from C_3_S, and the extent of the reaction increased. In addition, the high reaction heat influenced the product. Only calcite existed in the products, and no traces of aragonite and vaterite were detected. It is probably due to the high reaction heat, since calcite is the most stable phase among the three polymorphs of CaCO_3_ under high temperature [30].

**Figure 16:**
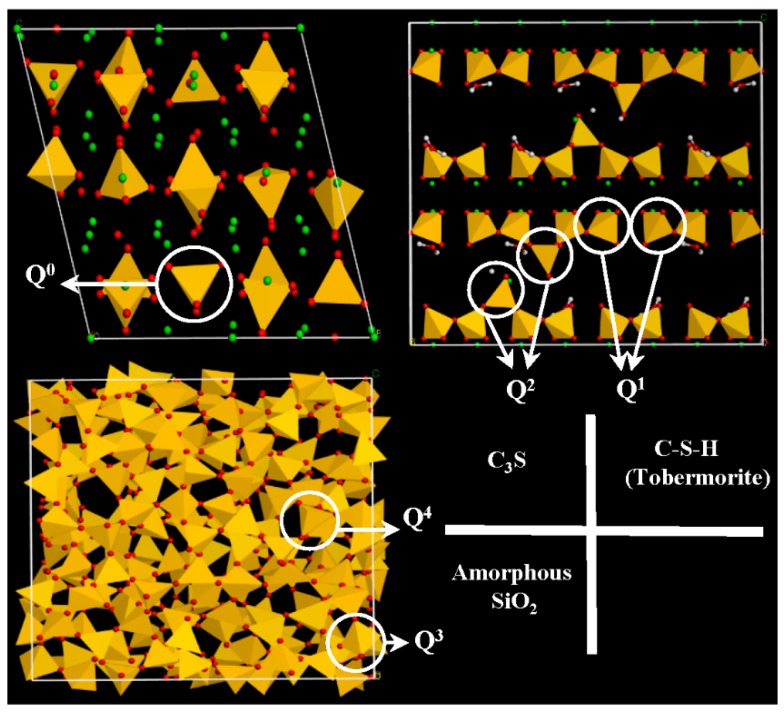
Molecular structure of C_3_S, C–S–H, amorphous SiO_2_, and different Q^n^ in their structure.

The TG-DTG, TEM-EDS, and ^29^Si MAS NMR results showed that there was only amorphous SiO_2_ and crystal CaCO_3_ in the carbonation products. The silicate phases reported by other researchers, such as C–S–H [9,10,11], calcium silicate hydrocarbonate [12], or Ca-modified silica gel [14], were not found in this study. Figure 16 shows the molecular structure of C_3_S, C–S–H, amorphous SiO_2_, and different Q^n^ in their structure. It can be seen from the figure that the Si in C_3_S belongs to Q^0^; Q^1^ and Q^2^ exist in C–S–H; and Q^3^ and Q^4^ exist in amorphous SiO_2_. From the perspective of the molecular structure, the cohesive force of C–S–H originated from the Van der Waals force and Coulomb force [31,32,33]. However, for amorphous SiO_2_, there is a three-dimensional network structure, leading to a Si–O bond for a higher bonding force. Hence, the amorphous SiO_2_ has a higher cohesive force than C–S–H.

**Figure 17:**
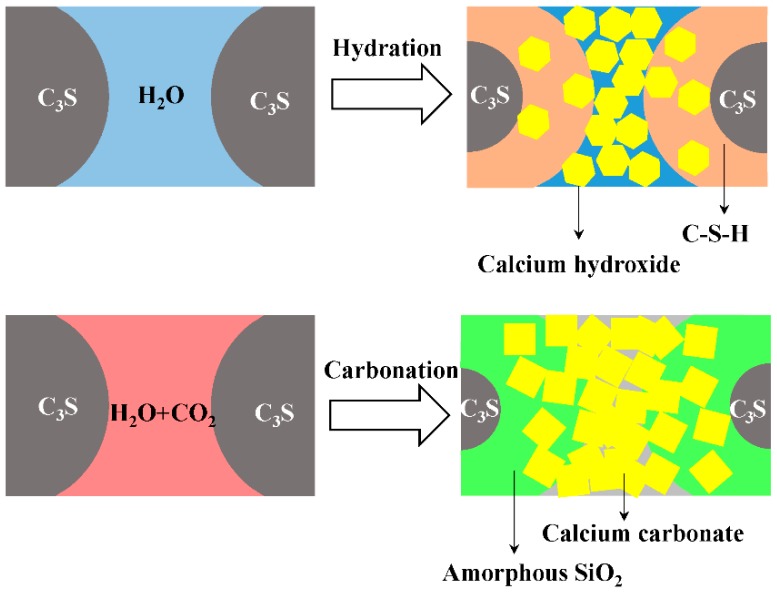
Schematic of the microstructure formation mechanism of C_3_S paste hydration and carbonation.

Figure 17 is a schematic diagram of the microstructure formation of C_3_S paste hydration and carbonation, which is based on former TEM-EDS results and other research results [34,35,36]. The main products of the hydration of C_3_S pastes are C–S–H and calcium hydroxide crystals. With the progress of a hydration reaction, the unreacted particles of C_3_S become smaller, while more hydration products develop [37]. Similar to the process of hydration, with the carbonation reaction proceeding, the size of the C_3_S particle decreases, while that of the carbonation products increases. The CaCO_3_ crystals that acted as a skeleton, and the amorphous SiO_2_ that acted as a matrix filling in the voids formed a dense structure, which significantly enhanced the strength. Compared with the hydration process, the carbonation reaction process is more rapid, and the reaction degree is higher. Therefore, the carbonation reaction is an accelerated hardening process.

## 5. Conclusions

In this study, the heat release characteristics and products of C_3_S paste subjected to carbonation curing were studied by micro-calorimetry, TG-DTG, XRD, TEM-EDS, and ^29^Si MAS NMR. The main conclusions drawn are as follows:

1. With the use of a micro-calorimeter under pressure of 0.5 bar, the maximum heat flow of C_3_S paste activated by carbonation was measured at 0.54 W/g. This is 200 times higher than that of the hydration reaction with the same w/b of 0.15. The carbonation heat of C_3_S is 116.7 kJ/mol, which is more than three times than that of hydration. It is indicative that the carbonation reaction is more rapid than the hydration for C_3_S paste with 0.15 w/b, and it also has a higher reaction degree than that in the hydration case. The experimental carbonation heat of C_3_S is lower than that of the calculated value. This is mainly because C_3_S does not completely react under experimental conditions.
2. Under 4-bar reaction pressure, the compressive strength of C_3_S paste reached 27.5 MPa by 2 h of carbonation, and 62.9 MPa by 24 h of carbonation. The compressive strength of the sample with 24 h of carbonation exceeded that of the 24-h hydration reference, and even exceeded that of the sample with 28 days of hydration.
3. Based on the TG-DTG data, the CO_2_ uptake by C_3_S paste carbonated for 2 h and 24 h reached 17.17% and 26.32%, respectively. This is evident that the materials containing C_3_S can have a high potential to absorb carbon dioxide through curing for the application of low-carbon products. It applies to Portland cement-based products.
4. The maximum CO_2_ uptake of C_3_S paste is tested under experimental conditions. Through the addition of water and repeated carbonation, the CO_2_ uptake of C_3_S paste can go up to 51.11%.
5. The carbonation products of C_3_S paste are mainly calcite crystal and amorphous SiO_2_, which is confirmed by TG-DTG, XRD, TEM-EDS, and ^29^Si MAS NMR. They are independent from carbonation degree and duration.

## Figures and Tables

**Table 1:** Chemical composition of synthesized C_3_S.

Chemical Composition	Content (%)
CaO	73.15
SiO_2_	26.06
Others	0.79

**Table 2:** Sample number and curing details.

Sample Number	Curing Conditions	Sample Form	Curing Age
H2h	Hydration curing; RH ≥ 95%; temperature = 20 °C.	Compact cylinder	2 h
H24h			24 h
H28d			28 days
C2h	Carbonation curing; purity of CO_2_ = 99.9%; curing pressure = 4 bar; RH ≥75%; temperature = 20 ± 3 °C.		2 h
C24h			24 h
RC		Powder	24 h × 4 times

**Table 3:** Weight loss, CO_2_ uptake, and carbonation degree of carbonation samples calculated by TG-DTG data.

Sample Number	Weight Loss (%)		CO_2_ Uptake (%)		Carbonation Degree (%)
	Decarbonation	Total	Experimental	Theoretical	
C2h	14.21	15.85	17.17	57.84	29.71
C24h	20.47	22.22	26.32		45.53
RC	31.28	34.89	51.11		88.43
